# Potential of AI and other tools for mitigating challenges associated with SCD, MCI or dementia in the workplace

**DOI:** 10.1002/alz70858_104857

**Published:** 2025-12-25

**Authors:** Emma Dixon, Elizabeth Gilman, Kandianos Emmanouil Sakalidis, Arlene Astell

**Affiliations:** ^1^ Clemson University, Clemson, SC, USA; ^2^ Northumbria University, Newcastle upon Tyne, United Kingdom

## Abstract

**Background:**

Individuals experiencing Subjective Cognitive Decline (SCD), Mild Cognitive Impairment (MCI) or early onset dementia face increased risk of unemployment or retirement earlier than planned. Providing workplace accommodations is one way to support this population to retain employment. Documented accommodations such as rotating employees to seemingly easier jobs or reducing their work hours, may cause further confusion and challenges for employees, ultimately resulting in loss of employment. This project aims to understand the experiences of people working with SCD, MCI and early onset dementia, the tools they utilize to continue in employment and opportunities for digital accommodations.

**Method:**

We conducted two rounds of one‐hour, semi‐structured interviews with 11 people with SCD, MCI and early onset dementia. The first session examined the declarative knowledge, procedural skills, and strategies that direct their work performance. In the second session we explored the task flows and critical points within those task flows for which participants would like support from assistive technology. We conducted reflexive thematic analysis of the transcribed interviews.

**Result:**

Participants in this study worked in diverse roles including lawyer, free‐lance writer, and therapist and divers industries, including manufacturing, engineering, and hospitality. In addition to outlining specific workplace challenges faced by participants in each of these roles, preliminary themes from our findings include: a) the eagerness and competency of participants to self‐explore emerging artificial‐intelligence (AI) tools to accommodate challenges in their work; b) the lack of in‐the‐moment adjustability of these AI tools to their fluctuating abilities; and c) use of these tools revealing the extent of someone's changes in abilities, amplifying self‐consciousness and leading to abandonment of potentially useful tools

**Conclusion:**

This study is uncovering ways people with SCD, MCI and early onset dementia working in diverse positions take initiative in identifying useful technologies to assist in their work. It is also revealing opportunities for existing tools to be modified to better meet their needs, as well as the personal impact of uptake of emerging AI tools.

